# Supplementary material for: Vitamin C protects retinal ganglion cells via SPP1 in glaucoma and after optic nerve damage
Source: Life Sci Alliance. 2023 May 9;6(8):e202301976. doi: 10.26508/lsa.202301976 (PMC10172762; doi:10.26508/lsa.202301976)
Supplement: Supplementary file 6 [file LSA-2023-01976_TableS1.docx]

**Table S1, Reagents and Resources**

| REAGENT or RESOURCE | | | SOURCE | | IDENTIFIER | | |
| --- | --- | --- | --- | --- | --- | --- | --- |
| Antibodies | | |  | |  | | |
| Mouse anti-BRN3A | | | Millipore | | Cat# MAB1585; RRID: AB_94166 | | |
| Chicken anti-GFAP | | | Abcam | | Cat# ab4674; RRID: AB_304558 | | |
| Mouse anti-Neurofilament H (SMI32) | | | BioLegend | | Cat# 801701; RRID: AB_2564642 | | |
| Goat anti-SPP1 | | | R&D Systems | | Cat# AF808; RRID: AB_2194992 | | |
| Biological Samples | | |  | | |  | |
| Mouse eye tissue | | | Mouse strain is listed in the  "Experimental Models:  Organisms/Strains" | | | N/A | |
| Chemicals, Peptides, and Recombinant Proteins | | |  | | |  | |
| HLM006474 (E2F1 inhibitor) | | | Tocris Bioscience | | | Cat# 5283 | |
| Retinoic acid | | | Tocris Bioscience | | | Cat# 0695 | |
| Ascorbic acid (vitamin C) | | | Sigma-Aldrich | | | Cat# A7506 | |
| Sodium ascorbate | | | Sigma-Aldrich | | | Cat# A7631 | |
| Experimental Models: Cell | | |  | | |  | |
| C57BL/6 astrocytes | | | This study | | | N/A | |
| Spp1 KO astrocytes | | | This study | | | N/A | |
| Experimental Models: Organisms/Strains | | |  | | |  | |
| Mouse: C57BL/6J (WT) | | | Jackson Laboratory | | | Cat# 000664 | |
| Mouse: B6.129S6(Cg)-*Spp1*^tm1Blh^/J | | | Jackson Laboratory | | | Cat# 004936 | |
| Mouse: B6.*Spp1*^fl-EGFP-stop-tdTomato^ | | | Produced by Cyagen | | | N/A | |
| Mouse: B6.Cg-Tg(*Gfap*-cre)77.6Mvs/2J | | | Jackson Laboratory | | | Cat# 024098 | |
| Mouse: B6.Cg-Gt(ROSA)26Sor^tm14(CAG-tdTomato)Hze^/J | | | Jackson Laboratory | | | Cat# 007914 | |
| Oligonucleotides | | |  |  | | | |
| Genotyping: Mouse B6.129S6(Cg)-*Spp1*^tm1Blh^/J forward | | | This study | 5'-GTCTGGAGAACATGGGTGCT-3' | | | |
| Genotyping: Mouse B6.129S6(Cg)-*Spp1*^tm1Blh^/J reverse | | | This study | 5'-GGGTGCAGGCTGTAAAGCTA-3' | | | |
| Genotyping: Mouse B6.*Spp1*^fl-EGFP-stop-tdTomato^ forward | | | This study | 5'-AGTGTAGAGCGTCATTGCAGTTT-3' | | | |
| Genotyping: Mouse B6.*Spp1*^fl-EGFP-stop-tdTomato^ reverse | | | This study | 5'-TCGGCTACATCCTAAATCCCATT-3' | | | |
| Genotyping: Mouse B6.Cg-Tg(*Gfap*-cre)77.6Mvs/2J forward | | | This study | 5'- TCCATAAAGGCCCTGACATC -3' | | | |
| Genotyping: Mouse B6.Cg-Tg(*Gfap*-cre)77.6Mvs/2J reverse | | | This study | 5'- TGCGAACCTCATCACTCGT-3' | | | |
| Genotyping: Mouse B6.Cg-Gt(ROSA)26Sor^tm14(CAG-tdTomato)Hze^/J forward (wt) | | | This study | 5'- AAGGGAGCTGCAGTGGAGTA -3' | | | |
| Genotyping: Mouse B6.Cg-Gt(ROSA)26Sor^tm14(CAG-tdTomato)Hze^/J reverse (wt) | | | This study | 5'- CCGAAAATCTGTGGGAAGTC -3' | | | |
| Genotyping: Mouse B6.Cg-Gt(ROSA)26Sor^tm14(CAG-tdTomato)Hze^/J forward (mut) | | | This study | 5'- CTGTTCCTGTACGGCATGG -3' | | | |
| Genotyping: Mouse B6.Cg-Gt(ROSA)26Sor^tm14(CAG-tdTomato)Hze^/J reverse (mut) | | | This study | 5'- GGCATTAAAGCAGCGTATCC -3' | | | |
| qPCR: *Spp1* forward | | | This study | 5'-AGCAAGAAACTCTTCCAAGCAA-3' | | | |
| qPCR: *Spp1* reverse | | | This study | 5'-GTGAGATTCGTCAGATTCATCCG-3' | | | |
| qPCR: *Runx1* forward | | | This study | 5'-GCCTTTTCCTTCCTGGTACTA-3' | | | |
| qPCR: *Runx1* reverse | | | This study | 5'-TCAGCGACTGTGTGCAGTGA-3' | | | |
| qPCR: *E2F1* forward | | | This study | 5'-CCCAACTACAAGCTGTGGATT-3' | | | |
| qPCR: *E2F1* reverse | | | This study | 5'-ATCCCGTCTGCACTCTCCT-3' | | | |
| qPCR: *Lcn2* forward | | | This study | 5'-CCGAAAGACTTCTGAAAACAAGC-3' | | | |
| qPCR: *Lcn2* reverse | | | This study | 5'-AAAGCGGGTGAAACGTTCCT-3' | | | |
| qPCR: *Steap4* forward | | | This study | 5'-CCCGAATCGTGTCTTTCCTA-3' | | | |
| qPCR: *Steap4* reverse | | | This study | 5'-GGCCTGAGTAATGGTTGCAT-3' | | | |
| qPCR: *S1pr3* forward | | | This study | 5'-AAGCCTAGCGGGAGAGAAAC-3' | | | |
| qPCR: *S1pr3* reverse | | | This study | 5'-TCAGGGAACAATTGGGAGAG-3' | | | |
| qPCR: *Timp1* forward | | | This study | 5'-AGTGATTTCCCCGCCAACTC-3' | | | |
| qPCR: *Timp1* reverse | | | This study | 5'-GGGGCCATCATGGTATCTGC-3' | | | |
| qPCR: *Hsbp1* forward | | | This study | 5'-GACATGAGCAGTCGGATTGA-3' | | | |
| qPCR: *Hsbp1* reverse | | | This study | 5'-GGATGGGGTGTAGGGGTACT-3' | | | |
| qPCR: *Cxcl10* forward | | | This study | 5'-CCCACGTGTTGAGATCATTG-3' | | | |
| qPCR: *Cxcl10* reverse | | | This study | 5'-CACTGGGTAAAGGGGAGTGA-3' | | | |
| qPCR: *Osmr* forward | | | This study | 5'-GTGAAGGACCCAAAGCATGT-3' | | | |
| qPCR: *Osmr* reverse | | | This study | 5'-GCCTAATACCTGGTGCGTGT-3' | | | |
| qPCR: *Aspg* forward | | | This study | 5'-GCTGCTGGCCATTTACACTG-3' | | | |
| qPCR: *Aspg* reverse | | | This study | 5'-GTGGGCCTGTGCATACTCTT-3' | | | |
| qPCR: *Gfap* forward | | | This study | 5'-CACGAACGAGTCCCTAGAGC-3' | | | |
| qPCR: *Gfap* reverse | | | This study | 5'-ATGGTGATGCGGTTTTCTTC-3' | | | |
| qPCR: *Vim* forward | | | This study | 5'-AGACCAGAGATGGACAGGTGA-3' | | | |
| qPCR: *Vim* reverse | | | This study | 5'-TTGCGCTCCTGAAAAACTGC-3' | | | |
| qPCR: *H2-D1* forward | | | This study | 5'-TCCGAGATTGTAAAGCGTGAAGA-3' | | | |
| qPCR: *H2-D1* reverse | | | This study | 5'-ACAGGGCAGTGCAGGGATAG-3' | | | |
| qPCR: *Serping1* forward | | | This study | 5'-TAGAGCCTTCTCAGATCCCGA-3' | | | |
| qPCR: *Serping1* reverse | | | This study | 5'-ACTCGTTGGCTACTTTACCCA-3' | | | |
| qPCR: *H2-T23* forward | | | This study | 5'-ACAGTCCCGACCCAGAGTAG-3' | | | |
| qPCR: *H2-T23* reverse | | | This study | 5'-CCACGTAGCCGACAATGATGA-3' | | | |
| qPCR: *Ggta1* forward | | | This study | 5'-GGTGGTTCCCAAGCTGGTTTA-3' | | | |
| qPCR: *Ggta1* reverse | | | This study | 5'-CGGGCGGTTCTTTGGATTGA-3' | | | |
| qPCR: *Iigp1* forward | | | This study | 5'-GGGGCAATAGCTCATTGGTA-3' | | | |
| qPCR: *Iigp1* reverse | | | This study | 5'-ACCTCGAAGACATCCCCTTT-3' | | | |
| qPCR: *Gbp2* forward | | | This study | 5'-CTGCACTATGTGACGGAGCTA-3' | | | |
| qPCR: *Gbp2* reverse | | | This study | 5'-GAGTCCACACAAAGGTTGGAAA-3' | | | |
| qPCR: *Fkbp5* forward | | | This study | 5'-TGAGGGCACCAGTAACAATGG-3' | | | |
| qPCR: *Fkbp5* reverse | | | This study | 5'-CAACATCCCTTTGTAGTGGACAT-3' | | | |
| qPCR: *Psmb8* forward | | | This study | 5'-ATGGCGTTACTGGATCTGTGC-3' | | | |
| qPCR: *Psmb8* reverse | | | This study | 5'-CGCGGAGAAACTGTAGTGTCC-3' | | | |
| qPCR: *Srgn* forward | | | This study | 5'-CTCGCCTTCGTCCTGGTTT-3' | | | |
| qPCR: *Srgn* reverse | | | This study | 5'-CCTCGATGCAGTTCGCAAAAA-3' | | | |
| qPCR: *Amigo2* forward | | | This study | 5'-GAGGCGACCATAATGTCGTT-3' | | | |
| qPCR: *Amigo2* reverse | | | This study | 5'-GCATCCAACAGTCCGATTCT-3' | | | |
| qPCR: *C3* forward | | | This study | 5'-CCAGCTCCCCATTAGCTCTG-3' | | | |
| qPCR: *C3* reverse | | | This study | 5'-GCACTTGCCTCTTTAGGAAGTC-3' | | | |
| qPCR: *Clcf1* forward | | | This study | 5'-GACTCGTGGGGGATGTTAGC-3' | | | |
| qPCR: *Clcf1* reverse | | | This study | 5'-CTAAGCTGCGGAGTTGATGCT-3' | | | |
| qPCR: *Ptx3* forward | | | This study | 5'-CCTGCGATCCTGCTTTGTG-3' | | | |
| qPCR: *Ptx3* reverse | | | This study | 5'-GGTGGGATGAAGTCCATTGTC-3' | | | |
| qPCR: *S100a10* forward | | | This study | 5'-TGGAAACCATGATGCTTACGTT-3' | | | |
| qPCR: *S100a10* reverse | | | This study | 5'-GAAGCCCACTTTGCCATCTC-3' | | | |
| qPCR: *Sphk1* forward | | | This study | 5'-ATGGAACCAGTAGAATGCCCT-3' | | | |
| qPCR: *Sphk1* reverse | | | This study | 5'-TCCGTTCGGTGAGTATCAGTTTA-3' | | | |
| qPCR: *Cd109* forward | | | This study | 5'-TCCCGCTTTCTGGTGACAG-3' | | | |
| qPCR: *Cd109* reverse | | | This study | 5'-ACCTGAGCCTTTACAAGGACC-3' | | | |
| qPCR: *Ptgs2* forward | | | This study | 5'-TTCAACACACTCTATCACTGGC-3' | | | |
| qPCR: *Ptgs2* reverse | | | This study | 5'-AGAAGCGTTTGCGGTACTCAT-3' | | | |
| qPCR: *Emp1* forward | | | This study | 5'-TTGGTGCTACTGGCTGGTCT-3' | | | |
| qPCR: *Emp1* reverse | | | This study | 5'-CATTGCCGTAGGACAGGGAG-3' | | | |
| qPCR: *Slc10a6* forward | | | This study | 5'-GGAGGGCCATGCGAATCTAAA-3' | | | |
| qPCR: *Slc10a6* reverse | | | This study | 5'-TGTCAGAGGCATAAGTCCAAAC-3' | | | |
| qPCR: *Tm4sf1* forward | | | This study | 5'-ACATCGGATACTCTCTGGTGTG-3' | | | |
| qPCR: *Tm4sf1* reverse | | | This study | 5'-TGGTCCTCCGTAGCATACTTT-3' | | | |
| qPCR: *B3gnt5* forward | | | This study | 5'-ATTCCCTGTCTCTCAAGCACA-3' | | | |
| qPCR: *B3gnt5* reverse | | | This study | 5'-GAACGTCGGCCATAGTTTTCA-3' | | | |
| qPCR: *Cd14* forward | | | This study | 5'-CTCTGTCCTTAAAGCGGCTTAC-3' | | | |
| qPCR: *Cd14* reverse | | | This study | 5'-GTTGCGGAGGTTCAAGATGTT-3' | | | |
| qPCR: *Gpc4* forward | | | This study | 5'-CTCAAGTCGAAAAGTTGCTCGG-3' | | | |
| qPCR: *Gpc4* reverse | | | This study | 5'-CTTCAAATGGTCACCGTTGATCT-3' | | | |
| qPCR: *Gpc6* forward | | | This study | 5'-CCAATCAGGCAGATTTGGACA-3' | | | |
| qPCR: *Gpc6* reverse | | | This study | 5'-GGGCCGAAAACGGGTGTTA-3' | | | |
| qPCR: *Sparc* forward | | | This study | 5'-GTGGAAATGGGAGAATTTGAGGA-3' | | | |
| qPCR: *Sparc* reverse | | | This study | 5'-CTCACACACCTTGCCATGTTT-3' | | | |
| qPCR: *Sparcl1* forward | | | This study | 5'-GGCAATCCCGACAAGTACAAG-3' | | | |
| qPCR: *Sparcl1* reverse | | | This study | 5'-TGGTTTTCTATGTCTGCTGTAGC-3' | | | |
| qPCR: *Thbs1* forward | | | This study | 5'-GGGGAGATAACGGTGTGTTTG-3' | | | |
| qPCR: *Thbs1* reverse | | | This study | 5'-CGGGGATCAGGTTGGCATT-3' | | | |
| qPCR: *Thbs2* forward | | | This study | 5'-CTGGGCATAGGGCCAAGAG -3' | | | |
| qPCR: *Thbs2* reverse | | | This study | 5'-GCTTGACAATCCTGTTGAGATCA-3' | | | |
| qPCR: *Il1a* forward | | | This study | 5'-CGCTTGAGTCGGCAAAGAAAT-3' | | | |
| qPCR: *Il1a* reverse | | | This study | 5'-CTTCCCGTTGCTTGACGTTG-3' | | | |
| qPCR: *Tnf* forward | | | This study | 5'-TGTGCTCAGAGCTTTCAACAA-3' | | | |
| qPCR: *Tnf* reverse | | | This study | 5'-CTTGATGGTGGTGCATGAGA-3' | | | |
| qPCR: *C1q* forward | | | This study | 5'-TCTGCACTGTACCCGGCTA-3' | | | |
| qPCR: *C1q* reverse | | | This study | 5'-CCCTGGTAAATGTGACCCTTTT-3' | | | |
| qPCR: *Megf10* forward | | | This study | 5'-CCCTCACTGTGCTGATAAATGT-3' | | | |
| qPCR: *Megf10* reverse | | | This study | 5'-TGATGGGGTTACACAAAGCTC-3' | | | |
| qPCR: *Mertk* forward | | | This study | 5'-CAGGGCCTTTACCAGGGAGA-3' | | | |
| qPCR: *Mertk* reverse | | | This study | 5'-TGTGTGCTGGATGTGATCTTC-3' | | | |
| qPCR: *Gas6* forward | | | This study | 5'-TGCTGGCTTCCGAGTCTTC-3' | | | |
| qPCR: *Gas6* reverse | | | This study | 5'-CGGGGTCGTTCTCGAACAC-3' | | | |
| qPCR: *Axl* forward | | | This study | 5'-ATGGCCGACATTGCCAGTG-3' | | | |
| qPCR: *Axl* reverse | | | This study | 5'-CGGTAGTAATCCCCGTTGTAGA-3' | | | |
| qPCR: *Tyro3* forward | | | This study | 5'-GCCTCCAAATTGCCCGTCA-3' | | | |
| qPCR: *Tyro3* reverse | | | This study | 5'-CCAGCACTGGTACATGAGATCA-3' | | | |
| qPCR: *Pros1* forward | | | This study | 5'-CGCTTTCGGGTGCTACTGG-3' | | | |
| qPCR: *Pros1* reverse | | | This study | 5'-CACTCTCGTTCAAGGTTGCC-3' | | | |
| qPCR: *Lrrk2* forward | | | This study | 5'-GCAATGCCTGCCTTACCTTCT-3' | | | |
| qPCR: *Lrrk2* reverse | | | This study | 5'-GGTGCTGATCTGATTCTTGCTAA-3' | | | |
| qPCR: *Ngf* forward | | | This study | 5'-GGCAGCTTTTTGGAAACTCCT-3' | | | |
| qPCR: *Ngf* reverse | | | This study | 5'-TTCACTGGCTTGAGGCACAG-3' | | | |
| qPCR: *Gdnf* forward | | | This study | 5'-AAGACCCACGTTTCGCATG-3' | | | |
| qPCR: *Gdnf* reverse | | | This study | 5'-TCCACTTCCCAGTCCTGCAG-3' | | | |
| qPCR: *Bdnf* forward | | | This study | 5'-AGGCACTGGAACTCGCAATG-3' | | | |
| qPCR: *Bdnf* reverse | | | This study | 5'-AAGGGCCCGAACATACGATT-3' | | | |
| qPCR: *Cdnf* forward | | | This study | 5'-CTTTTGCGCCGGGTTTTGTAT-3' | | | |
| qPCR: *Cdnf* reverse | | | This study | 5'-AGGGAGTTGTAGAATCGGTCTAA-3' | | | |
| qPCR: *Manf* forward | | | This study | 5'-TCTGGGACGATTTTACCAGGA-3' | | | |
| qPCR: *Manf* reverse | | | This study | 5'-TCTTGCTTCACGGCAAAACTTTA-3' | | | |
| qPCR: *Lif* forward | | | This study | 5'-ATTGTGCCCTTACTGCTGCTG-3' | | | |
| qPCR: *Lif* reverse | | | This study | 5'-GCCAGTTGATTCTTGATCTGGT-3' | | | |
| qPCR: *Nrtn* forward | | | This study | 5'-GGGCTACACGTCGGATGAG-3' | | | |
| qPCR: *Nrtn* reverse | | | This study | 5'-CCAGGTCGTAGATGCGGATG-3' | | | |
| qPCR: *Pspn* forward | | | This study | 5'-GGCAGATAAGCTCTCATTTGGG-3' | | | |
| qPCR: *Pspn* reverse | | | This study | 5'-CACAGTCGGCATGAACCAG-3' | | | |
| qPCR: *Artn* forward | | | This study | 5'-CCCTAGCTGTTCTAGCCCTG-3' | | | |
| qPCR: *Artn* reverse | | | This study | 5'-AGGGTTCTTTCGCTGCACAA-3' | | | |
| qPCR: *Fgf1* forward | | | This study | 5'-CCCTGACCGAGAGGTTCAAC-3' | | | |
| qPCR: *Fgf1* reverse | | | This study | 5'-GTCCCTTGTCCCATCCACG-3' | | | |
| qPCR: *Aph1b* forward | | | This study | 5'-TGGTCGTCATAATGCTGCACG-3' | | | |
| qPCR: *Aph1b* reverse | | | This study | 5'-CACCAGATGCGTCAGGAGA-3' | | | |
| qPCR: *Aph1c* forward | | | This study | 5'-CCTGTGTTCTTCGGTTGCG-3' | | | |
| qPCR: *Aph1c* reverse | | | This study | 5'-CATGGACGAAAGCAGGAGAGA-3' | | | |
| qPCR: *Atp5d* forward | | | This study | 5'-CATGTCCCCACACTACAGGTC-3' | | | |
| qPCR: *Atp5d* reverse | | | This study | 5'-TCGGCATTCACAGTGACG-3' | | | |
| qPCR: *Atp5g1* forward | | | This study | 5'-AGTTGGTGTGGCTGGATCA-3' | | | |
| qPCR: *Atp5g1* reverse | | | This study | 5'-GCTGCTTGAGAGATGGGTTC-3' | | | |
| qPCR: *Atp5g2* forward | | | This study | 5'-ACCCCTGAAAATGTACGCC-3' | | | |
| qPCR: *Atp5g2* reverse | | | This study | 5'-TCTCATCTGTTGGCATCTGTG-3' | | | |
| qPCR: *Atp5g3* forward | | | This study | 5'-GGAGAGGGCTCTACAGTTTTTA-3' | | | |
| qPCR: *Atp5g3* reverse | | | This study | 5'-TGGCAGCAGTATCAATGTCTC-3' | | | |
| qPCR: *Atp5l* forward | | | This study | 5'-GAGAAGGCACCGTCGATGG-3' | | | |
| qPCR: *Atp5l* reverse | | | This study | 5'-ACACTCTGAATAGCTGTAGGGAT-3' | | | |
| qPCR: *Bace2* forward | | | This study | 5'-GGAGCCTGTCAGGGCTACT-3' | | | |
| qPCR: *Bace2* reverse | | | This study | 5'-CCACAAGAATCTGTACCTTCTGC-3' | | | |
| qPCR: *Cox5a* forward | | | This study | 5'-GCCGCTGTCTGTTCCATTC-3' | | | |
| qPCR: *Cox5a* reverse | | | This study | 5'-GCATCAATGTCTGGCTTGTTGAA-3' | | | |
| qPCR: *Cox6a1* forward | | | This study | 5'-TCAACGTGTTCCTCAAGTCGC-3' | | | |
| qPCR: *Cox6a1* reverse | | | This study | 5'-AGGGTATGGTTACCGTCTCCC-3' | | | |
| qPCR: *Cox6b2* forward | | | This study | 5'-CAGAACCAGACGCGTAACTGCT-3' | | | |
| qPCR: *Cox6b2* reverse | | | This study | 5'-GCTGATGGGACACAGTGAATGG-3' | | | |
| qPCR: *Cox7a1* forward | | | This study | 5'-GCTCTGGTCCGGTCTTTTAGC-3' | | | |
| qPCR: *Cox7a1* reverse | | | This study | 5'-GTACTGGGAGGTCATTGTCGG-3' | | | |
| qPCR: *Cox7a2* forward | | | This study | 5'-GCTGGCCCTTCGTCAGATT-3' | | | |
| qPCR: *Cox7a2* reverse | | | This study | 5'-GGCATCCCATTATCCTCCTGAA-3' | | | |
| qPCR: *Gpx7* forward | | | This study | 5'-TCCGAGCAGGACTTCTACGAC-3' | | | |
| qPCR: *Gpx7* reverse | | | This study | 5'-TCTCCCTGTTGGTGTCTGGTT-3' | | | |
| qPCR: *Maob* forward | | | This study | 5'-ATGAGCAACAAAAGCGATGTGA-3' | | | |
| qPCR: *Maob* reverse | | | This study | 5'-TCCTAATTGTGTAAGTCCTGCCT-3' | | | |
| qPCR: *Ndufa11* forward | | | This study | 5'-GGTGAAGCGGTTCTTTGAATCT-3' | | | |
| qPCR: *Ndufa11* reverse | | | This study | 5'-GAGCCGATTATGCCGCAAATG-3' | | | |
| qPCR: *Ndufa12* forward | | | This study | 5'-ACCGATGGGTCATCTACACCA-3' | | | |
| qPCR: *Ndufa12* reverse | | | This study | 5'-TCGTCAGTCATGCAGTGAAGC-3' | | | |
| qPCR: *Ndufa3* forward | | | This study | 5'-TCCACCCGGAAGTCATCAG-3' | | | |
| qPCR: *Ndufa3* reverse | | | This study | 5'-GCACCTCTTGTGTGTGGTGA-3' | | | |
| qPCR: *Ndufa4* forward | | | This study | 5'-TCCCAGCTTGATTCCTCTCTT-3' | | | |
| qPCR: *Ndufa4* reverse | | | This study | 5'-GGGTTGTTCTTTCTGTCCCAG-3' | | | |
| qPCR: *Ndufa5* forward | | | This study | 5'-GCTGAAAAAGAACTAAGTCTGGC-3' | | | |
| qPCR: *Ndufa5* reverse | | | This study | 5'-CATATTGGCCACTTCCACTG-3' | | | |
| qPCR: *Ndufb11* forward | | | This study | 5'-CCTCCAGGGCTGTAATCGC-3' | | | |
| qPCR: *Ndufb11* reverse | | | This study | 5'-GGTTCTTCGCGTAGACGTTTTC-3' | | | |
| qPCR: *Ndufb2* forward | | | This study | 5'-CCCCGGTACAGGGAGTTTC-3' | | | |
| qPCR: *Ndufb2* reverse | | | This study | 5'-GCCAAAATCGCCAAAGAATCCA-3' | | | |
| qPCR: *Ndufb6* forward | | | This study | 5'-ATAACTTTTTGCGGGACGGG-3' | | | |
| qPCR: *Ndufb6* reverse | | | This study | 5'-CAGGAAAATCTCTCATTGGTG-3' | | | |
| qPCR: *Ndufs4* forward | | | This study | 5'-CTGCCGTTTCCGTCTGTAGAG-3' | | | |
| qPCR: *Ndufs4* reverse | | | This study | 5'-TGTTATTGCGAGCAGGAACAAA-3' | | | |
| qPCR: *Prkn* forward | | | This study | 5'-GTCACAAGACTCAACGATC-3' | | | |
| qPCR: *Prkn* reverse | | | This study | 5'-TGCTCTTCTCCAAGGATC-3' | | | |
| qPCR: *Snca* forward | | | This study | 5'-ACCAAAGAGCAAGTGACAAATGT-3' | | | |
| qPCR: *Snca* reverse | | | This study | 5'-TGTCCAGGGTTATTACTGAGCA-3' | | | |
| qPCR: *Ucp2* forward | | | This study | 5'-ATGGTTGGTTTCAAGGCCACA-3' | | | |
| qPCR: *Ucp2* reverse | | | This study | 5'-CGGTATCCAGAGGGAAAGTGAT-3' | | | |
| qPCR: *Uqcr10* forward | | | This study | 5'-ATCCCTTCGCGCCTGTACT-3' | | | |
| qPCR: *Uqcr10* reverse | | | This study | 5'-GTGCTCGTAGATCGCGTCT-3' | | | |
| qPCR: *Xdh* forward | | | This study | 5'-ATGACGAGGACAACGGTAGAT-3' | | | |
| qPCR: *Xdh* reverse | | | This study | 5'-TCATACTTGGAGATCATCACGGT-3' | | | |
| qPCR: *Cox11* forward | | | This study | 5'-GTGCCCCTCTATCGGCTCTA-3' | | | |
| qPCR: *Cox11* reverse | | | This study | 5'-TAATGACCCGGTCCTTGACAG-3' | | | |
| qPCR: *Cox6c* forward | | | This study | 5'-GCGTCTGCGGGTTCATATTG-3' | | | |
| qPCR: *Cox6c* reverse | | | This study | 5'-TCTGCATACGCCTTCTTTCTTG-3' | | | |
| qPCR: *Ndufaf1* forward | | | This study | 5'-TTTGTGGGTAGTCACTGTGTAGA-3' | | | |
| qPCR: *Ndufaf1* reverse | | | This study | 5'-TGTCCTTGTAAGCCCTCTTCAG-3' | | | |
| qPCR: *Sdha* forward | | | This study | 5'-GGAACACTCCAAAAACAGACCT-3' | | | |
| qPCR: *Sdha* reverse | | | This study | 5'-CCACCACTGGGTATTGAGTAGAA-3' | | | |
| qPCR: *Uqcrb* forward | | | This study | 5'-GGCCGATCTGCTGTTTCAG-3' | | | |
| qPCR: *Uqcrb* reverse | | | This study | 5'-CATCTCGCATTAACCCCAGTT-3' | | | |
| qPCR: *Cox6b1* forward | | | This study | 5'-ACTACCTGGACTTCCACCG-3' | | | |
| qPCR: *Cox6b1* reverse | | | This study | 5'-ACCCATGACACGGGACAGA-3' | | | |
| qPCR: *Gapdh* forward | | | This study | 5'-GGTTGTCTCCTGCGACTTCAA-3' | | | |
| qPCR: *Gapdh* reverse | | | This study | 5'-CCTGTTGCTGTAGCCGTATTCAT-3' | | | |
| Software and Algorithms | |  | | | | |  |
| GraphPad Prism 8 | https://www.graphpad.com/ | | | | | | RRID: SCR_002798 |
| Adobe Photoshop CS6 | https://www.adobe.com/products/photoshop.html | | | | | | RRID:SCR_014199 |
| Fiji | https://fiji.sc/ | | | | | | RRID:SCR_002285 |
| BioRender | https://www.biorender.com | | | | | | RRID:SCR_018361 |

**Resource availability**

**Lead Contact**

Lead contact is Tatjana C. Jakobs ([tatjana_jakobs@meei.harvard.edu](mailto:tatjana_jakobs@meei.harvard.edu)).

**Materials Availability**

Further information and requests for resources and reagents in this study are available upon reasonable request to lead contact with a completed Materials Transfer Agreement.

**Data Availability**

All data are available in the main text or the supplementary materials.
